# An Unusual Outbreak of Ochratoxicosis Associated with *Trigonella foenum-graecum* Ingestion in Ruminants from Different Farms of Sicily

**DOI:** 10.3390/toxins17030120

**Published:** 2025-03-02

**Authors:** Maria Rizzo, Patrizia Licata, Pietro Paolo Niutta, Michela Pugliese, Vito Macaluso, Giovanna Lucrezia Costa, Giuseppe Bruschetta, Fabio Bruno

**Affiliations:** 1 Department of Veterinary Sciences, University of Messina, Via Palatucci Annunziata, 98168 Messina, Italy; 2 Independent Researcher, 90121 Palermo, Italy

**Keywords:** clinical signs, mycotoxin, ochratoxin a, ruminants, Sicily, *Trigonella foenum-graecum*

## Abstract

*Trigonella foenum-graecum* is a widely cultivated legume in Mediterranean regions, and it is used for human and animal consumption, as well as for medical purposes. High temperatures and abundant rainfall during the spring season in Sicily favor the formation of an environment suitable for the growth and proliferation of fungi with the production of mycotoxins. In this study, ochratoxin A, aflatoxin, deoxynivalenol, zearalenone, fumonisin, and T-2 toxin concentrations in *Trigonella foenum-graecum* were determined in feed administered to ruminants and also in blood samples from cattle and sheep in order to evaluate the toxicity correlated to the possible presence of these mycotoxins based on the clinical signs observed in the animals. Analyses of mycotoxins in fenugreek and blood samples were conducted using the enzyme immunoassay KIT. Five extensive farms sited in the northwest of the Sicily region, with a total of 90 intoxicated animals, reported a concomitant unusual outbreak of neurological disorders. Decreased spinal reflex responses, postural abnormalities associated with weakness or recumbency, and hyperesthesia of the limbs suggested a problem regarding the peripheral nervous system. The mortality rate recorded was very high, even reaching 100% of the intoxicated animals. OTA intoxication in Sicilian ruminants represents an important warning on the vulnerability of farms to mycotoxin contamination and underlines the importance of preventive measures and monitoring in animal health management.

## 1. Introduction

In Sicily, fenugreek (*Trigonella foenum-graecum*, Fabaceae) is a widely cultivated legume, similarly to other Mediterranean regions, as well as Yemen and India. It is used for human and animal consumption, as well as for medical purposes [1]. The leaves (in dried or fresh form) and seeds (used as spices) of this plant are used in the production of hormones (steroids), other molecules for pharmaceutical use, and in the nutraceutical industry as additives for artificial flavorings and supplements in wheat and maize flour [2,3]. Although fenugreek is generally considered to be safe as an edible plant, different toxicity studies conducted in rodents and rabbits have revealed its potential ability to cause harmful effects [1,4,5]. Fenugreek is indicated for therapeutic purposes in the treatment of a variety of medical conditions, including diabetes, cancer, hyperlipidemia, inflammation, neurotoxicity, hepatotoxicity, ulcers, wounds, bacterial and fungal infections, leg weakness, and edema [6,7]. Green fenugreek leaves are a rich source of β-carotene (19 mg/100 g), ascorbic acid (220 mg/100 g), dietary fiber, iron, calcium, and zinc [8]. Fenugreek seeds are a rich source of dietary fiber (50–65 g/100 g), mainly non-starch polysaccharides [9]. The endosperm is rich in proteins (43.8 g/100 g): globulin, lecithin, and albumin [10,11]. It has a high percentage of free amino acids (20–30%), especially 4-hydroxyisoleucine and histidine, which can stimulate insulin activity [12]. The seeds contain 5.5–7.5% total lipids, composed primarily of neutral lipids (85%), phospholipids (10%), and glycolipids (5%). The plant is also a valuable source in livestock feed due to the presence of bioactive components, including flavonoids, polyphenols, and proanthocyanidins, which enhance the biological and nutritional value of the fodder by demonstrating a good antioxidant capacity [13]. Forages, like cereals and other plant species, are vulnerable to fungal infections and mycotoxin contamination [14]. The tropical and subtropical climatic conditions of Sicily, characterized by high temperatures and abundant precipitation during the spring season, promote the formation of an environment suitable for the growth and proliferation of molds, resulting in the subsequent production of mycotoxins [15]. Ochratoxin A (OTA) is a mycotoxin produced by *Aspergillus ochraceus* and *Penicillium verrucosum*, contaminating various agricultural products like wheat, maize, coffee, and dried fruit [16]. Monogastric animals can experience health problems when exposed to the toxin, while cattle are more resistant to OTA intoxication due to their ruminal microbiota [17]. The presence of OTA in feed intended for ruminant consumption is generally not a cause for significant concern due to the potential detoxification capacity of ruminants and the low concentration of OTA in forages [18]. Long-term exposure can cause diseases such as Balkan nephropathy (BEN), nephropathy in pigs, hepatotoxicity, immunotoxicity, and even cancer [19]. OTA’s mechanism of action is related to the formation of adducts with DNA macromolecules, which permits the classification of this toxin as a potential Group 2B human carcinogen [20,21]. The presence of OTA in feed is typically the result of the inadequate drying of cereals and unsatisfactory hygienic storage conditions [22]. The toxin’s effects depend on the animal species, age, diet, and the growth conditions of the fungus [23]. In response to public health concerns, the Commission Regulation (EU) 2023/915 has set a limit of 10 µg/kg for OTA in dried herbs [24]. Mycotoxins in animal feed also include aflatoxins, deoxynivalenol, zearalenone, fumonisins, and T-2 toxins [25]. Aflatoxin is classified as a Group 1 carcinogen (IARC), the ingestion of which is known to cause various types of multi-organ damage, including hepatotoxicity, immunosuppression, coagulation impairment, anorexia, hemorrhage, edema, and jaundice. Reduced weight gain is one of the direct effects of aflatoxin poisoning. In dairy cattle, contamination can lead to a decrease in milk production and lower milk quality. Aflatoxins also have negative effects on the reproductive system of ruminants, leading to reduced reproductive performance, such as decreased fertility and increased rates of spontaneous abortion [26]. Deoxynivalenol (DON) is a mycotoxin produced mainly by *Fusarium graminearum,* which acts on animal cells, blocking protein synthesis and causing high gastrointestinal toxicity, vomiting, and immunodeficiency [27,28]. Chronic exposure to DON can negatively impact growth rates and reduce reproductive efficiency, especially in younger animals and pregnant females. The effects of DON are known to cause anorexia or reduced feed intake in ruminants. This can lead to poor weight gain and decreased productivity [27,28]. Zearalenone (ZEA) are toxins produced by *Fusarium graminearum* and *Fusarium verticilliodes*. They cause immunotoxicity, hyperestrogenism, reproductive toxigenicity with prolapses, and abortions due to ZEA [29,30]. The estrogenic effects of ZEA can interfere with the normal growth and development of young ruminants, leading to reduced weight gain and overall growth performance. Moreover, ZEA can suppress immune function, making ruminants more vulnerable to infections and diseases [29,30]. Fumonisins (FUMs) are mycotoxins produced by *Fusarium proliferatum.* Fumonisins have been shown to cause neurological effects in ruminants, particularly affecting the brain. Their immunosuppressive effects can negatively impact livestock health, especially in young or stressed animals. Moreover, cattle are particularly susceptible to liver damage when fumonisin contamination in their feed is high [29,30].

T-2 and HT-2 toxins are mycotoxins belonging to the trichothecenes family and they are produced by various species of fungi, including *Fusarium*, *Cephalosporium* and *Trichoderma.* T-2 toxins can cause severe irritation to the mucous membranes of the mouth, esophagus, and gastrointestinal tract in ruminants. Due to the toxic effects on the gastrointestinal system, immune system, and overall metabolic functions, T-2 toxins can lead to reduced growth and weight gain in ruminants. In severe cases, this can affect production rates, such as milk yield in dairy cows and weight gain in beef cattle. In ruminants, neurological symptoms such as uncoordinated movement, tremors, and even convulsions may occur in cases of high exposure. These effects can severely impact the animal’s ability to function normally and reduce its overall performance [31].

This study aimed to determine the concentrations of ochratoxin A, aflatoxin, deoxynivalenol, zearalenone, fumonisins, and T-2 toxin in samples of *Trigonella foenum-graecum* administered as feed in cattle and sheep showing neurological signs suggestive of intoxication.

## 2. Results

**Figure 1:**
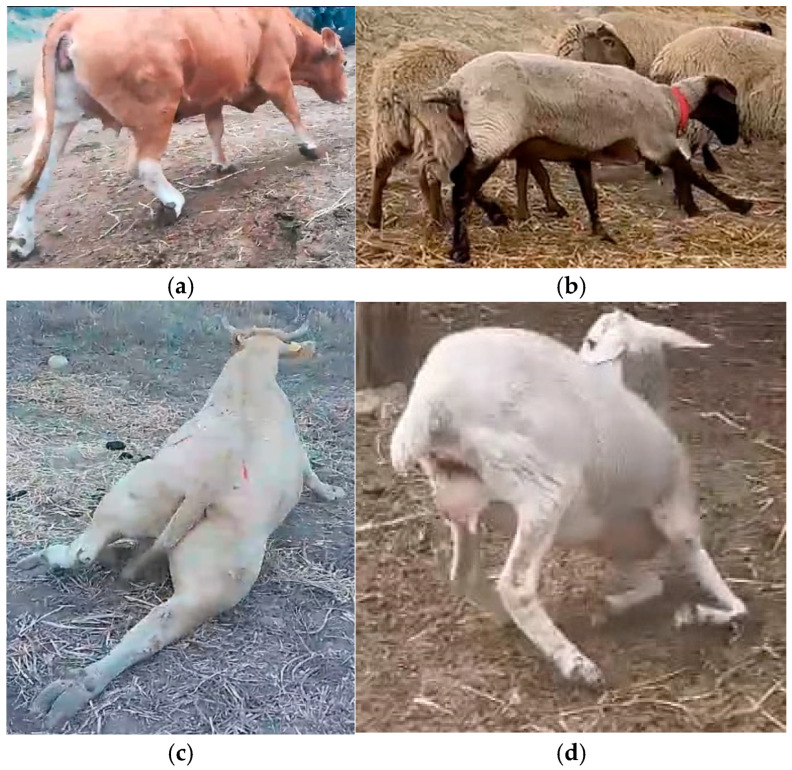
Fenugreek intoxication in cattle and sheep. (**a**) Intoxicated cattle knuckled over in their hindlimb fetlocks. (**b**) Intoxicated sheep with ataxic gait and hypermetria. (**c**) Intoxicated cattle in recumbency and hypotonia of hindlimbs. (**d**) Intoxicated sheep in a genuflecting position.

Animals showed specific neurological deficits such as loss of conscious proprioception, diminishing or absent patellar reflexes, triceps, flexor, panniculus, and anal sphincter reflexes, and hypotonia (Figure 1a–d; Table 1).

The development from the onset of clinical signs to recumbency ranged from 3 days to a few weeks for cattle and from 10 to 15 days for sheep. Despite the neurological disorders, they appeared bright and alert. No modifications were reported regarding feeding and drinking.

The decreased spinal reflex response, the postural abnormalities associated with weakness or recumbency, and the hyperesthesia of the limbs suggested a problem regarding the peripheral nervous or muscular system.

The hematological parameters evaluated did not show significant modifications, except a moderate leukocytosis associated with neutrophilia in a low percentage (8%) of animals affected that did not seem related to clinical signs. An increase from moderate to high level in ALP, AST, LDH, and CK was detected in 47.3% of sheep and 23.4% of cows. Table 2 and Table 3 summarize the results of the hematological and biochemical parameters assayed.

A supportive therapy including B-complex vitamins (B1, B2, B3, B6, and B12; Vit D3), minerals (calcium and magnesium salts), and non-steroidal anti-inflammatory drugs (tolfenamic acid, flunixin meglumin) was administered without an improvement in symptoms. The affected animals died spontaneously. Four sheep which were pregnant at the time of the onset of clinical signs died spontaneously after giving birth to healthy offspring.

Different causes of peripheral nervous and/or muscular system involvement were postulated and examined. Based on clinical history revealing the consumption of fenugreek straw concurrently with the onset of neurological signs, intoxication was suspected.

The ochratoxin A concentrations found in *Trigonella foenum-graecum* ranged from a minimum of 21.25 ppb to a maximum of 23.16 ppb (Table 4). However, the concentrations of aflatoxins, deoxynivalenol, zearalenone, fumonisins, and T-2 toxins were below the limit of detection (LOD).

Ochratoxin A values in the sheep serum samples analyzed showed values between 10.5 and 18.38 ppb. The OTA values found in bovine serum for animals intended for milk production showed values between 0.2 and 0.8 ppb, while in beef cows, the concentrations ranged from values of 1.61 to 4.57 ppb.

## 3. Discussion

Given the risk of a potential transfer from feed to animal tissues and products, legislative authorities have issued or proposed regulations for the control of OTA in food and/or feed. In 2006, the Commission of the European Communities [24] recommended that Member States ensure compliance with the guidance values indicated for the acceptability of compound feed, cereals, and cereal products for animal feed. The values vary according to feed and animal species.

In many areas of Sicily with a strong zootechnical vocation, both extensive and intensive, it is possible to come across pathological situations that can refer to intoxications and/or poisonings of this type. The absence of specific scientific studies does not facilitate the task of veterinarians, as certain and “certified” news from publications is scarce and fragmentary. Our study is the first, as far as we know, to be published in Italy on the toxic effects resulting from the intake of *Trigonella foenum-graecum* in cattle and sheep. Some brief considerations may be made, as follows:

– Climate change is inducing changes in agronomic choices in vast regions and, among these, in Sicily above all; these changes include an increase in atmospheric temperature, which causes aridity and abrupt meteorological changes, sudden rains, and, often, floods. In this context of changes, the choice of using *Trigonella foenum graecum* suits our territory quite well, as it is a very resistant plant; however, with a certain frequency, we see the wetting of freshly dried hay that is baled in the presence of high humidity. This can lead to the development of fungi and therefore the presence of mycotoxins in these forages;
– In Sicily, we are witnessing a change in livestock breeding: many dairy farms have transformed into meat farms. Even in areas particularly suited to milk production, such as the Ragusa area, farms that include cow–calf lines of Limousine breed animals, often purebred or crossed with Charolaise meat breeds have sprung up;
– The two conditions that the five cattle and sheep breeding farms have in common are the same type of fodder used for feeding and similar clinical signs highlighted in the animals;
– In the considered farms, not all animals were administered *Trigonella foenum graecum,* and the appearance of symptoms occurred only in animals that had consumed this hay; what we found is comparable to what was noted in a study conducted in Spain Moreno B. et al., (2023), confirming that neurological signs appeared only in animals that had consumed fenugreek [32];
– In studies by Lustig M. (1958) and Adler J.He and Egyed M. (1961) in Israel [33,34], Bourke CA (2009) in Australia [35], and Moreno B. et al. (2023) in Spain, there is no mention of the finding of mycotoxins in fenugreek [32]. Even so, only Moreno B. et al. (2023) reported looking for total aflatoxins;
– None of the researchers who described clinical signs related to fenugreek intake tried to clarify which pathogenesis could be called into question in the determination of the pathology. Any toxic components present in the structure of the plant in question (*Trigonella foenum graecum*) or present in the animal organism and of vegetal “derivation” are not identified or hypothesized. For this reason, having detected the presence of Ochratoxin A in the hay leads us to believe that this toxin plays a significant role in the onset of the pathology. Another hypothesis could be linked to the presence of high values of Ochratoxin A, which could be favored by the specific substrate represented by fenugreek, leading to an amplification of the toxic mechanisms in the animal organism; therefore, we could hypothesize that there is a synergy between any toxic components of the plant that have not yet been highlighted and Ochratoxin A.

The toxic effects of OTA on goats have also been shown since, in the 1970s, Harwig et al. (1975) reported that the intravenous infusion of OTA at a dose of 1 mg/kg body weight caused the death of sheep within 24 h [36]. The potentially harmful effects of OTA are mainly related to the prolonged ingestion of a contaminated diet; this has led several researchers to study the degree of the detoxification of OTA in the digestive tract and the amount of OTA in the blood of sheep. Such studies include Xiao et al. (1991), who conducted both an in vitro and in vivo study to determine the extent of OTA hydrolysis in the rumen in sheep fed diets with different ratios of cereals and hay [37]. Since sheep, cattle, and goats can degrade OTA in the rumen, acute poisoning seems to be an uncommon event; however, it must be emphasized that rumen functionality represents the main barrier to the passage of the toxin into the bloodstream; therefore, correct feeding constitutes the main preventive action against ochratoxicosis. In our study, the mortality rate recorded was very high, even reaching 100% of the intoxicated animals in the farms affected, and this is strongly correlated with economic losses for farmers given the serious impairment of the nervous system of the animals, which, in the last stages of the disease, were unable to maintain a quadrupedal position.

It has been reported that fenugreek straw can be the cause of nervous disorders in cattle and sheep, leading to the degeneration of the peripheral nerves of the limbs. The clinical presentation of intoxication may appear based on the species considered. Indeed, in cattle, it is described a progressive paresis involving the hindlimbs, while in sheep, the nervous disorder affects the forelimbs, which is associated with a final involvement of the central nervous system [32,34,35,38,39,40]. The clinical presentation of the neurological disorder may vary in relation to the stage of the disease. In the end stage, intoxicated animals usually tend to knuckle over their fetlocks until terminal recumbency. The appearance of clinical signs is strongly related to the amount of fenugreek consumed. It has been reported that, as in the present cases, a percentage of between 8 and 12% of animals present clinical signs after ingestion [35], in partial accordance with data reported herein, where the percentage of animals affected ranged between 18.2 and 65%. Similarly, the onset of clinical signs is also strongly related to the consumption of fenugreek; in natural conditions, it ranges between 11 days and 6 weeks for sheep [35,38] and 4–8 weeks for cows, in which the signaling of natural intoxication occurrence is episodic [32,33].

The diagnosis of intoxication of fenugreek in cows and sheep may result hard, given that other, more common disorders must be considered in the differential diagnosis; these include mineral and vitamin deficits (e.g., hypocalcemia, hypokalemia, hypomagnesemia, or vitamin E deficiency) and chemical (e.g., organophosphates) and plant poisoning (e.g., Cycadales) [41,42].

Experimental studies conducted on rodents reported that fenugreek ingestion can determine reproductive disorders such as hormonal alterations, abortions, and late development of the nervous system in litter [5]. Sheep that were pregnant before becoming intoxicated gave birth to healthy and viable lambs prior to death. Fenugreek intoxication is not lethal to animals. In 2023, Moreno et al. reported a complete recovery after 6 months in an experimental study on sheep, suggesting that fenugreek intoxication may be reversible [32]. However, it is important to prevent the formation of mycotoxins through the use of pesticides and improved hay preservation methods in order to reduce the risk of contamination in feed intended for livestock.

## 4. Conclusions

The outbreak of ochratoxin A poisoning in *Trigonella foenum graecum*, affecting cattle and sheep in Sicily, represents an unprecedented event in Italy, as it is the first documented case in our country. Ochratoxin A, a mycotoxin produced by fungi such as *Aspergillus* and *Penicillium,* is well known for its toxic effects on various organs, such as the liver and kidneys, but in these cases of intoxication, it caused peripheral neuropathy in ruminants. This study showed that fenugreek can be a source of ochratoxin A contamination, with toxic effects on cattle and sheep. It is essential to monitor forage quality, especially in humid environments, where fungal growth can be favored. The intoxication led to neurological disorders, such as muscle weakness and motor difficulties, typical signs of damage to the peripheral nervous system. This is new compared to other cases of ochratoxin A poisoning, which usually focus on kidney or liver damage. This case highlights the importance of implementing rigorous monitoring of mycotoxins in animal feed, especially hay, which is a critical feed for ruminants. Preventive farming practices, such as the use of fungicides and improved hay storage techniques, could reduce the risk of contamination.

However, further studies into the link between ochratoxin A and neuropathy in ruminants are necessary, as this case could be a sign of other risks that are not yet recognized. In addition, there is a need to raise awareness among farmers and health authorities about the possible contamination of hay and how to control these risks.

In conclusion, the ochratoxin A intoxication in Sicilian ruminants represents an important warning regarding the vulnerability of farms to mycotoxin contamination and underlines the importance of preventive measures and monitoring in animal health management.

## 5. Materials and Methods

### 5.1. Animals and Clinical Signs

A total of five extensive farms located in the northwest of the Sicily region reported a concomitant unusual outbreak of neurological disorders. The farms reared beef cows (n.3), with a total of 50 intoxicated animals, dairy cows (n.1), with 21 sick bovines, and sheep (n.1), with 19 poisoned ruminants. The cattle farms were formed of different breeds, including Limousine and Charolaise for beef cattle and Friesian for dairy cattle. The sheep farm reared Suffolk sheep and crossbreeds. The animals were kept in open-sided housing within a partially covered area and had access to a large outdoor paddock. The incidence of the animals affected ranged between 18.8 and 50%. Animals were regularly vaccinated against common endemic livestock diseases; also, a systematic control program against endo- (intestinal nematodes) and ectoparasites (ticks, mites, lice, flies, and myiasis) was applied. The clinical signs exhibited that were detected in affected animals involved ataxia, hypermetria, posterior limb ataxia progressing to generalized paresis, and recumbency in cattle, while in sheep, only the forelimbs were affected. A neurological examination was performed in all animals presenting neural clinical signs according to a standardized protocol. Animals were examined following a standardized procedure including the evaluation of mentation, behavior, gait, posture, balance, proprioception, spinal reflexes and musculature, cranial nerve signs, definite or possible cranial nerve involvement, and pain signaling [43].

### 5.2. Hematological and Biochemical Evaluation

Based on the clinical history and neurological examination, serum samples were collected from fasting animals with neurological disorders to perform proper hematological and biochemical tests.

Hematological and biochemical parameters were measured within 1–2 h of serum sample collection from fasting animals that presented neurological disorders. Hematology was performed using a hematology analyzer based on focused flow impedance and flow cytometry (ProCyte Dx, Idexx Laboratories, Westbrook, ME, USA) calibrated for bovine and sheep values. Glucose (GLU), total cholesterol (CHOL), triglyceride (TG), total protein (TP), albumin (ALB), creatinine (CREA), and urea blood concentrations were detected using commercial kits provided by the manufacturer (SPINREACT, St. Esteve de Bas, Spain), and a spectrophotometer (A560, Fulltech, Rome, Italy). Enzymes such as aspartate aminotransferase (AST), creatine kinase (CPK), lactate dehydrogenase (LDH), and alkaline phosphatase (ALP) were analyzed using commercial kits (SPINREACT, St. Esteve de Bas, Spain) and a spectrophotometer (A560, Fulltech, Rome, Italy) [44]. For all biochemical parameters, intra- and inter-assay CVs were between 0.5% and 3.2% and between 2.9% and 4.5%, respectively.

### 5.3. OTA Determination in Serum Samples

Health status was verified by clinical history, physical examination, and blood tests. Ruminant farmers signed a voluntary informed consent form prior to blood tests being performed on the animals. A total of 5 mL of blood per animal was drawn from the jugular veins of seventy-one cows and nineteen sheep. Blood samples were placed in individual sampling tubes and stored at −20 °C until analysis. The determination of OTA was carried out with an enzyme immunoassay kit, RIDASCREEN Ochratoxin A 30/15, R-Biopharm AG, Darmstadt, Germany, with some changes to the protocol utilized. A total of 3 ml of serum sample was extracted by shaking for 7 min using 5 mL of n-hexane and 3.0 mL of HCl. Then, the sample was centrifugated for 25 min at 2500× *g* at 12 °C and the supernatant was removed. The N-hexane stratum was filtered and 3 mL was extracted with 3 mL of NaHCO_3_ buffer (0.15 M, pH 8.5), and the samples was centrifuged for 8 min at 2800× *g* at 12 °C. The NaHCO_3_ buffer double stratum was incorporated and extracted with 3 mL of n-hexane and 1 mL of HCl and after mixed and centrifuged for 7 min, 2800× *g*, at 12 °C. Buffer layers were eliminated and the obtained extracts were dried with a nitrogen stream and reconstituted with 1 mL NaHCO_3_ buffer. Finally, the samples were analyzed with ELISA method.

### 5.4. Trigonella foenum-graecum Sample Collection

Two hundred fifty-five samples of *Trigonella foenum-graecum* were obtained from five sheep and cattle farms (four for meat production, cow–calf line, and one for milk production) located in the Bisacquino area, near Palermo, Sicily. The feed provided to these animals was exclusively hay derived from *Trigonella foenum-graecum*. For each hay bale, three samples were collected from the central, middle, and outer regions of the bale. The samples were stored in a cool place protected against light. After the extraction, all samples were analyzed in duplicate on 96-well multiplates according to the mycotoxin kit manufacturer’s protocol (R-Biopharm AG, Pfungstadt, Germany). Samples (fenugreek mixture from the three bale zones) were taken from each bale, carefully ground, and mixed before extraction.

### 5.5. Determination of Ochratoxin A

Analyses for ochratoxins were conducted using an enzyme immunoassay kit for the quantitative analysis of ochratoxin A, RIDASCREEN Ochratoxin A 30/15, R-Biopharm AG, Germany, according to the manufacturer’s protocol. The ground samples were weighed (10 g) separately and then homogenized with an appropriately diluted extraction solution (ECO extractor 10×). The mixture was shaken for 6 min and the obtained solutions were centrifuged for 5 min at 3500× *g* at 23 °C. After the sample extraction, the test involved the evaluation of the reaction between the antigen and antibody in wells coated with antibodies against ochratoxin A, in accordance with the manufacturer’s protocol. From the extraction solutions obtained, 1 mL of supernatant was recovered and diluted with 1 mL of wash buffer solution; then, it was used to proceed with the actual ochratoxin A assay by adding 50 μL of the diluted supernatants to the wells of the 96-multiplate. The addition of ochratoxin A standards (0 μg/L, 0.03 μg/L, 0.1 μg/L, 0.3 μg/L, 1 μg/L, 3 μg/L) and the extracted solution samples, as well as the enzyme conjugate, enables free and enzyme-conjugated ochratoxin A to compete for the specific binding sites located on the antibodies according to the principles of a competitive enzyme immunoassay process. After dark incubation (30 min at 23 °C), the unbound enzyme conjugates were removed in the subsequent wash steps. The addition and subsequent incubation (15 min at 23 °C) with the substrate/chromogen allowed the bound enzyme conjugate to convert the substrate/chromogen into a blue reaction product. The stop solution interrupted the reaction, resulting in a color change from blue to yellow. The final step was the spectrophotometric measurement, performed at 450 nm using a BIORAD 680 plate reader (BIORAD Laboratories, Segrate, Italy).

### 5.6. Determination of Aflatoxins

The evaluation of aflatoxins was assessed by an enzyme immunoassay kit for the quantitative determination of aflatoxins (B1, B2, G1, G2), EuroProxima Total Aflatoxin, R-Biopharm AG, Germany, according to the manufacturer’s protocol. The ground samples were weighed (3 g) separately and then homogenized with the extraction solution (80% methanol). The samples were shaken and centrifuged for 10 min at 2000× *g* at 23 °C. The extraction solutions obtained were used to recover 50 μL of the supernatant to dilute with 150 μL of dilution buffer; then, it was used to proceed with the assay by adding 50 μL of the diluted supernatants to each well. The test involved the evaluation of the reaction between the antigen and antibody in wells coated with aflatoxin antibodies. The addition of aflatoxin standards (0.00625 ng/mL, 0.0125 ng/mL, 0.025 ng/mL, 0.05 ng/mL, 0.1 ng/mL, 0.2 ng/mL) and the extracted samples, with the addition of the enzyme conjugate, allowed for the competitive reaction for the binding sites of the antibodies. After dark incubation (1 h at 37 °C), the unbound enzyme conjugates were removed in the subsequent wash steps. The addition and following incubation (30 min at 23 °C) with the substrate/chromogen and the addition of the stop solution allowed for the spectrophotometric measurement, performed at 450 nm using a BIORAD 680 plate reader (BIORAD Laboratories, Italy).

### 5.7. Determination of Deoxynivalenol

Deoxynivalenol concentrations were obtained through the use of an enzyme immunoassay kit for the quantitative determination of deoxynivalenol, RIDASCREEN DON, R-Biopharm AG, Germany, according to the manufacturer’s protocol. The ground samples were weighed (5 g) separately and then homogenized with the extraction solution (25 mL dH_2_O). The samples were vortexed and shaken for 3 min, and the obtained solutions were filtered (Whatman 1). Then, the solutions were used to recover 50 μL of the supernatant from each well; we then proceeded with the assay in a coated plate with specific anti-deoxynivalenol antibodies, in accordance with the manufacturer’s protocol. The deoxynivalenol standards (0 ppb, 3.7 ppb, 11.1 ppb, 33.3 ppb, 100 ppb) or sample solutions were added to the wells to start the reaction with the binding sites of the antibodies. After dark incubation (30 min at 23 °C), the unbound enzyme conjugates were removed with subsequent wash steps, and another incubation (15 min at 23 °C) was performed with the substrate/chromogen. The absorption values were assessed at 450 nm using a BIORAD 680 plate reader (BIORAD Laboratories, Italy).

### 5.8. Determination of Zearalenone

The concentrations of zearalenone was obtained from the samples by means of an enzyme immunoassay kit for the quantitative determination of Zearalenone, RIDASCREEN Zearalenon, R-Biopharm AG, Germany, according to manufacturer’s protocol. The ground samples were weighed (5 g) separately and then mixed with extraction solution (25 mL of methanol/water (70/30), vortexed, shaken for 3 min and centrifuged for 10 min 3500× *g* at 23 °C. The solutions obtained were used to recover 100 μL of the supernatant and dilute it with 600 μL of the dilution buffer in order to proceed with the assay by adding 50 μL of the diluted supernatant to each well. The sample solution obtained by extraction, together with zearalenone standards (0 ppt, 50 ppt, 150 ppt, 450 ppt, 1350 ppt, 4050 ppt), was used for the reaction with anti-zearalenone antibodies coated in the multiplates’ wells. After dark incubation (2 h at 23 °C), the unbound enzymes were removed in subsequent wash steps, and the addition of the substrate/chromogen with following incubation (30 min at 23 °C) allowed for the conversion of the substrate/chromogen into a blue reaction product. The stop solution interrupted the reaction, resulting in a color change from blue to yellow in order to perform the spectrophotometric measurement at 450 nm using a BIORAD 680 plate reader (BIORAD Laboratories, Italy).

### 5.9. Determination of Fumonisins

Fumonisins were assayed using an enzyme immunoassay kit for the quantitative determination of fumonisins, RIDASCREEN Fumonisin ECO, R-Biopharm AG, Germany, according to the manufacturer’s protocol. The ground samples used for extraction were weighed (5 g) and mixed with a properly diluted extraction buffer (ECO extractor 10×). The samples were vortexed and shaken for 6 min. Then, samples were centrifuged for 3 min and 3500× *g* at 23 °C. From the obtained solutions, 100 μL of the supernatant was added to 900 μL of the sample dilution buffer to proceed with the assay by then adding 50 μL of the diluted supernatant to each well. The solutions obtained, together with fumonisin standards (0 μg/mL, 0.025 µg/mL, 0.075 µg/mL, 0.222 µg/mL, 0.666 µg/mL, 2 µg/mL), were used to proceed with the reaction with anti-fumonisin antibodies. After dark incubation (30 min at 23 °C), with subsequent wash steps and another incubation (15 min at 23 °C) with the substrate/chromogen, the addition of the stop solution interrupted the reaction in order to perform spectrophotometric measurements at 450 nm using a BIORAD 680 plate reader (BIORAD Laboratories, Italy).

### 5.10. Determination of T-2 Toxins

T-2 Toxins were obtained by the use of an enzyme immunoassay kit for the quantitative determination of T-2 Toxin, RIDASCREEN T-2 Toxin, R-Biopharm AG, Germany, according to the manufacturer’s protocol. The ground samples were weighed (5 g) and added to a diluted extraction solution (25 mL methanol/dH_2_O 70/30 (*v*/*v*), vortexed for 3 min, and centrifuged for 10 min at 3500× *g* at 23 °C. The supernatant (100 μL) was recovered and diluted with the sample buffer (600 µL). An additional dilution was obtained with 50 µL of diluted extract and 450 µL of PBS buffer solution containing methanol 10%. The extracts were used to proceed with the assay by adding 50 μL of the diluted supernatants to each well. The test involved the evaluation of the reaction between the antigen and antibody in wells coated with specific T-2 Toxin antibodies. The addition of T-2 Toxin standards (0 µg/L, 0.1 µg/L, 0.2 µg/L, 0.4 µg/L, 0.8 µg/L, 1.6 µg/L) or sample solutions, as well as the addition of enzyme conjugate, enabled free and enzyme-conjugated T-2 Toxin to compete for the specific binding sites located on the antibodies. After dark incubation (1 h at 23 °C), the unbound enzyme conjugates were removed from the antibodies in the wash steps. The addition of the substrate/chromogen and the following incubation (30 min at 23 °C) allowed the enzyme conjugate to convert the substrate/chromogen into a blue reaction product, and the addition of the stop solution to interrupt the reaction resulted in a color change from blue to yellow. Absorbance was recorded at a wavelength of 450 nm using a BIORAD 680 plate reader (BIORAD Laboratories, Italy).

### 5.11. Statistical Analysis

G Power 3.1 software was used to determine the sample size for an “a priori” ANOVA test (fixed effect, omnibus, one-way), with an effect size (f) of 0.25, a significance level (α) of 0.05, a power (1 − β) of 0.90, and five groups. Statistical analysis was performed using GraphPad Prism 9.0 (GraphPad Software, Inc., Boston, MA, USA). The Shapiro–Wilk normality test was performed, and data were reported as mean ± SD. Differences among samples were assessed by one-way ANOVA followed by the Bonferroni test, and statistical significance was set at *p* < 0.05.

## Figures and Tables

**Table 1:** Data of farms, breed of ruminants, and intoxicated and dead animals.

Farm	Farm Production	Breed	Total Animals Reared (n.)	Total Animals Intoxicated (n.)	Total Animals Intoxicated (%)	Total Dead (n.)	Total Dead (%.)
A	Sheep	Crossbreed and Suffolk	102	19	18.6	19	100
B	Beef Cows	Limousine	28	11	39.3	11	100
C	Beef Cows	Crossbreed and Limousine	143	26	18.2	26	100
D	Dairy Cows	Friesian	110	21	19.1	21	100
E	Beef Cows	Charolaise	20	13	65	13	100

**Table 2:** Hematological evaluation performed on blood samples of cattle and sheep showing neurological disorders.

Hematological Parameters	Unit	Cows		Sheep	
		RI	Intoxicated	RI	Intoxicated
Red blood cells	10^6^/μL	5.0–10.0	6.5 ± 1.9	7.3–11.3	8.5 ± 2.1
Hematocrit	%	28–38	32 ± 4	29–38	34 ± 2.1
Hemoglobin	g/dL	9–14	11 ± 2.4	8–12	9.1 ± 1.6
White blood cells	10^3^/μL	4.0–10.0	9.56 ± 5	4.0–10.0	8.6 ± 0.3.42
Neutrophils	10^3^/μL	1.0–3.5	2.84 ± 1.56	0.7–4.0	2.62 ± 1.17
Lymphocytes	10^3^/μL	2.5–5.5	3.4 ± 1.2	2.0–4.0	2.6 ± 1.8
Monocytes	10^3^/μL	0–0.33	0.1 ± 0.2	0–0.7	0.1 ± 0.3
Eosinophils	10^3^/μL	0.3–1.5	0.54 ± 0.41	0.1–1.0	0.04 ± 0.32
Basophils	10^3^/μL	0–0.1	0.01 ± 0.03	0–0.3	0.01 ± 0.02
Platelets	10^3^/μL	300–800	585 ± 77	200–800	376 ± 65

**Table 3:** Biochemical evaluation performed on blood samples of cattle and sheep showing neurological disorders.

Biochemical Parameters	Unit	Cows		Sheep	
		RI	Intoxicated	RI	Intoxicated
GLU	mg/dL	68–104	75.6 ± 8.9	50–80	65.6 ± 7.2
CHOL	mg/dL	163–220	189 ± 36	43–103	124 ± 26
TG	mg/dL	10–19	14 ± 3.2	5.3–30	13.2 ± 9.5
TP	g/dL	6.7–8.8	7.8 ± 0.8	6.0–7.9	6.9 ± 0.7
ALB	g/dL	3.3–4.3	3.7 ± 0.4	2.4–3.0	2.8 ± 0.3
CREA	mg/dL	0.6–1.4	1.0 ± 0.2	1.2–1.9	1.6 ± 0.2
Urea	mg/dL	7–19	14 ± 5	10–35	19 ± 7.9
AST	IU/L	54–135	100.13 ± 96.43	60–280	156 ± 139.8
CK	IU/L	88–292	241.3 ± 87.2	8–100	70 ± 38.1
LDH	IU/L	725–1122	993 ± 267	238–440	273 ± 185
ALP	IU/L	27–127	114.4 ± 89.5	70–390	252.8 ± 136.4

RI = reference interval; GLU = glucose; CHOL = total cholesterol; TG = triglyceride; TP = total protein; ALB = albumin; CREA = creatinine; Urea = urea; AST = aspartate aminotransferase; CK = creatine kinase; LDH = lactate dehydrogenase; ALP = alkaline phosphatase.

**Table 4:** Ochratoxin A detected in samples of *Trigonella foenum-graecum* round bales collected from farms located in the Bisacquino area.

	Farms				
Sampled Round Bales	A	B	C	D	E
1	21.016	22.034	22.749	21.242	22.703
2	21.635	21.651	22.427	21.489	24.053
3	20.223	21.615	22.521	21.505	24.040
4	22.732	21.631	21.974	20.608	23.259
5	21.428	21.411	22.206	20.793	22.449
6	21.712	21.945	22.072	21.318	23.015
7	21.707	21.316	22.111	21.437	23.388
8	20.832	21.236	22.250	21.376	22.438
9	20.892	21.575	22.312	21.667	22.738
10	20.907	21.424	22.430	21.401	23.366
11	22.130	22.129	22.013	21.444	23.232
12	21.852	21.635	22.531	20.829	23.054
13	21.227	21.681	22.518	20.819	23.208
14	21.023	21.683	22.600	21.709	23.705
15	20.851	21.489	22.705	21.639	22.839
16	22.243	21.578	22.604	20.773	23.404
17	20.918	22.188	22.397	21.137	22.899
Mean ± SD	21.37 ± 0.6383 *^α^	21.66 ± 0.2720 **^βγ^**	22.38 ± 0.2405 **^δε^**	21.25 ± 0.3533 **^ζ^**	23.16 ± 0.4780

* Differences between farms A and C; ^α^ differences between farms A and E; ^β^ differences between farms B and C; ^γ^ differences between farms B and E; ^δ^ differences between farms C and D; ^ε^ differences between farms C and E; ^ζ^ differences between farms D and E (*p* < 0.001). Data are expressed as mean or mean ± standard deviation.

## Data Availability

The original contributions presented in this study are included in this article. Further inquiries can be directed to the corresponding author.
